# BioCARS: Synchrotron facility for probing structural dynamics of biological macromolecules

**DOI:** 10.1063/4.0000238

**Published:** 2024-01-31

**Authors:** Robert W. Henning, Irina Kosheleva, Vukica Šrajer, In-Sik Kim, Eric Zoellner, Rama Ranganathan

**Affiliations:** BioCARS, Center for Advanced Radiation Sources, The University of Chicago, Chicago, Illinois 60637, USA

## Abstract

A major goal in biomedical science is to move beyond static images of proteins and other biological macromolecules to the internal dynamics underlying their function. This level of study is necessary to understand how these molecules work and to engineer new functions and modulators of function. Stemming from a visionary commitment to this problem by Keith Moffat decades ago, a community of structural biologists has now enabled a set of x-ray scattering technologies for observing intramolecular dynamics in biological macromolecules at atomic resolution and over the broad range of timescales over which motions are functionally relevant. Many of these techniques are provided by BioCARS, a cutting-edge synchrotron radiation facility built under Moffat leadership and located at the Advanced Photon Source at Argonne National Laboratory. BioCARS enables experimental studies of molecular dynamics with time resolutions spanning from 100 ps to seconds and provides both time-resolved x-ray crystallography and small- and wide-angle x-ray scattering. Structural changes can be initiated by several methods—UV/Vis pumping with tunable picosecond and nanosecond laser pulses, substrate diffusion, and global perturbations, such as electric field and temperature jumps. Studies of dynamics typically involve subtle perturbations to molecular structures, requiring specialized computational techniques for data processing and interpretation. In this review, we present the challenges in experimental macromolecular dynamics and describe the current state of experimental capabilities at this facility. As Moffat imagined years ago, BioCARS is now positioned to catalyze the scientific community to make fundamental advances in understanding proteins and other complex biological macromolecules.

## INTRODUCTION

The basic properties of proteins—folding, binding, catalysis, signal transmission, and allosteric regulation—all emerge from a global pattern of forces acting between all constituent atoms. Because these properties require great precision in the positioning and dynamics of amino acids, it is common to speak of proteins as if they are finely tuned machines that are precisely arranged for mediating their selected functions. In an influential review,^1^ Bruce Alberts makes the point that we call proteins “machines” because *“…like the machines invented by humans to deal efficiently with the macroscopic world, these protein assemblies contain highly coordinated moving parts. Within each protein assembly, intramolecular collisions are not only restricted to a small number of possibilities, but reaction C depends on reaction B, which in turn depends on reaction A—just as it would in a machine of our common experience….”* This insightful exposition of the low-dimensional, concerted nature of amino acid dynamics within proteins is indeed borne out in empirical observations. The conformational cycling of proteins and protein complexes between a small number of macroscopic states underlies many biological processes, for example, DNA replication,^2^ metabolism,^3,4^ transport,^5^ cellular motility,^6^ and signal processing.^7^ Thus, the biology of proteins is rooted in their collective mechanics: the motions and fluctuations that define the functional reaction coordinate and the forces constraining those dynamics. As in man-made machines, a comprehensive description of the internal dynamics is the key to explaining how structure leads to function.^8^

However, the analogy with man-made devices is incomplete and perhaps even misleading. Unlike conventional machines, proteins are the product of an evolutionary process that produces marginally stable structures with the capacity for high-performance functions. For example, the native state of even diffusion-limited “perfect enzymes”^9^ can display a net free energy separating the folded and unfolded states of just −8 kcal/mol^10^ equivalent to about three decent hydrogen bonds—distributed over all the atomic interactions that make up the tertiary structure! It has been argued that such marginal stability is essential for the dynamics underlying function^11^ and for the capacity to evolve.^12^ The coexistence of high-performance with plasticity required by functional and evolutionary dynamics implies that strong heterogeneity and cooperativity exist in the pattern of internal forces that underlie the “design” of evolved proteins.^13,14^ Indeed, catalytic specificity in proteases,^15^ signal transmission within G-protein-coupled receptors,^16^ the cooperative binding of oxygen molecules to hemoglobin,^17^ catalysis in the metabolic enzyme dihydrofolate reductase,^18^ and antigen recognition by antibody molecules^19,20^ all depend on the concerted action of a few strongly coupled amino acids that are distributed both near to and far from the active site. These “hotspot” residues seem to be embedded within an overall environment of neutral interactions.^21,22^ These findings imply that though the amino acid dynamics defining the reaction coordinate might be low-dimensional, collective, and machine-like as envisioned by Alberts, this functional mechanism is likely to be embedded in a sea of high-dimensional, local, and possibly irrelevant motions. Such a heterogeneous design is distinct from that of engineered machines (where high performance usually depends on global optimization of constraints) but might be the natural solution for evolved systems.^23^ With regard to timescales, the implication is that we should expect a broad range of possible motions within proteins from the scale of atomic vibrations (picoseconds) to many seconds or longer, with the relevant motions distributed in some yet unknown way. Thus, we know that proteins are in some sense, machines, but we do not know what sort of machines they are, and what principles underlie their design in light of their evolutionary origin.

These observations frame the conceptual and practical challenges in developing effective technologies for studying macromolecular dynamics. The marginal stability of native states and the steep distance and geometry dependence of the fundamental forces acting between atoms mean that free energy changes along a protein reaction coordinate can be associated with very subtle, small scale structural movements. Thus, we must use methods that can deliver sub-angstrom atomic resolution. The heterogeneity and cooperativity of amino acid interactions means that we need methods that can sample a broad range of time-scales, including the slower regimes (hundreds of nanoseconds to seconds and longer) where collective actions of amino acids are expected to be concentrated.^24–26^ Since many motions are likely to be irrelevant, we need analytic approaches to isolate the biologically relevant motions and to connect them to the organization of physical forces between atoms. Finally, we need generality, such that the core technologies for experimental molecular dynamics are applicable to all biological macromolecules and not just restricted to special cases.

By these criteria, many current biophysical methods are insufficient. Nuclear magnetic resonance spectroscopy provides information about the chemical environment of nuclear spins and changes in this environment during dynamical fluctuations^27^ and has provided key insights about weakly populated excited states.^3,28^ However, restriction in time scales of measurements, the inability to directly visualize collective motions, and the difficulty of relating the measured parameters to physical forces limits our progress. Single molecule force spectroscopy can relate global conformational transitions to applied external forces,^29^ but does not provide the atomic detail necessary to define the origins of these transitions in the internal mechanics.

These considerations represent the basis for strategic focus of the BioCARS facility. Time-resolved x-ray crystallography (TRX) offers a direct route to observing a broad range of motions with high temporal and spatial resolution.^30^ Importantly, classic work shows that many proteins continue to carry out their biochemical activities in the crystal state,^31–36^ a finding that has been reinforced in many studies.^37–42^ Indeed, using various techniques for starting reactions within proteins, TRX has provided detailed information about local and collective motions associated with the reaction coordinate(s) on relevant timescales.^43–46^ In recent years, these experiments have been brought to a level of sophistication, robustness, and generality that opens up dynamical studies of many biological macromolecules. A complementary and widely used method is time-resolved x-ray solution scattering (TRXSS), an experimental modality that exposes a wealth of global information about motions and conformational heterogeneity in proteins, also on a broad range of time scales.^47,48^ In the context of a stable, professional environment to conduct these studies, TRX and TRXSS have the potential to transform our understanding of protein mechanics. In this review, we present the history and current state of the BioCARS synchrotron facility, with particular focus on the nontrivial technical capabilities—many imagined by Keith Moffat and implemented under his leadership—which make these difficult experiments possible.

## HISTORY OF THE FACILITY

BioCARS is a national user facility located at Sector 14 of the Advanced Photon Source at Argonne National Laboratory. It has been supported primarily by NIH since its founding in 1992. It was originally designed and built for operating two bending magnet stations (14-BM-C and 14-BM-D) and one insertion device station (14-ID). Initial operation involved standard, static crystallography using monochromatic radiation in all three experimental stations as well as time-resolved crystallography using polychromatic radiation (Laue diffraction) on 14-ID beamline. From the beginning though, the main scientific focus at BioCARS was the development of the time-resolved crystallography technique for studies of structural dynamics of biological macromolecules. As BioCARS PI for 26 years, Moffat pioneered this technique as a tool for capturing biomolecules in action at atomic resolution and in real time, as they perform their function. He made a number of essential contributions to the development of this field. Early efforts toward the ability to record such molecular movies started while Moffat was at MacCHESS, Cornell University in the 1980s.^49–52^ Moffat then moved to the University of Chicago to use the opportunities enabled by the new, high brilliance Advanced Photon Source (APS), a third-generation synchrotron source and to establish BioCARS synchrotron facility at the APS dedicated to protein dynamics. The early, proof-of-principle milliseconds and nanoseconds time-resolved crystallography experiments were conducted by Moffat and colleagues at the NSLS and ESRF synchrotron facilities while BioCARS facility was being designed and built.^53–58^ First time-resolved studies resulting from experiments conducted at BioCARS were published in the early 2000s.^59–66^

One limitation of the original BioCARS 14-ID insertion device beamline design was that single 100 ps pulse isolation could only be performed during the so-called “hybrid” mode of the APS storage ring. This was typically limited to only 2 weeks per APS run cycle so a limited number of experiments could be performed with this time resolution. Even with this limitation, the technique was successfully demonstrated, and a time-resolved crystallography community was developed. The success of the time-resolved program led to an upgrade of the beamline in 2007–2008^67^ to improve the x-ray flux (dual undulators), focusing (new mirrors) and to be able to isolate single x-ray pulses from the standard 24-bunch mode of operation at the APS (new x-ray chopper). This greatly expanded the number of time-resolved experiments that could be performed and, along with a new picosecond laser system installed at the same time, extended the shortest time-resolution from nanoseconds to 100 ps.

Since this upgrade, 14-ID beamline is almost exclusively used for time-resolved studies, while bending magnet stations, used in the past for static monochromatic crystallography, are no longer being used. Although the 14-ID beamline was originally designed for time-resolved crystallography, increased interest in time-resolved solution scattering in the user community and very high polychromatic flux available at the beamline led us to explore conducting simultaneous time-resolved SAXS/WAXS experiments as well.^68^ Infrastructure for this technique was developed in the late 2000s. Today, each program uses about 50% of the available user beamtime.

## 14-ID BEAMLINE DESCRIPTION

**FIG. 1.**
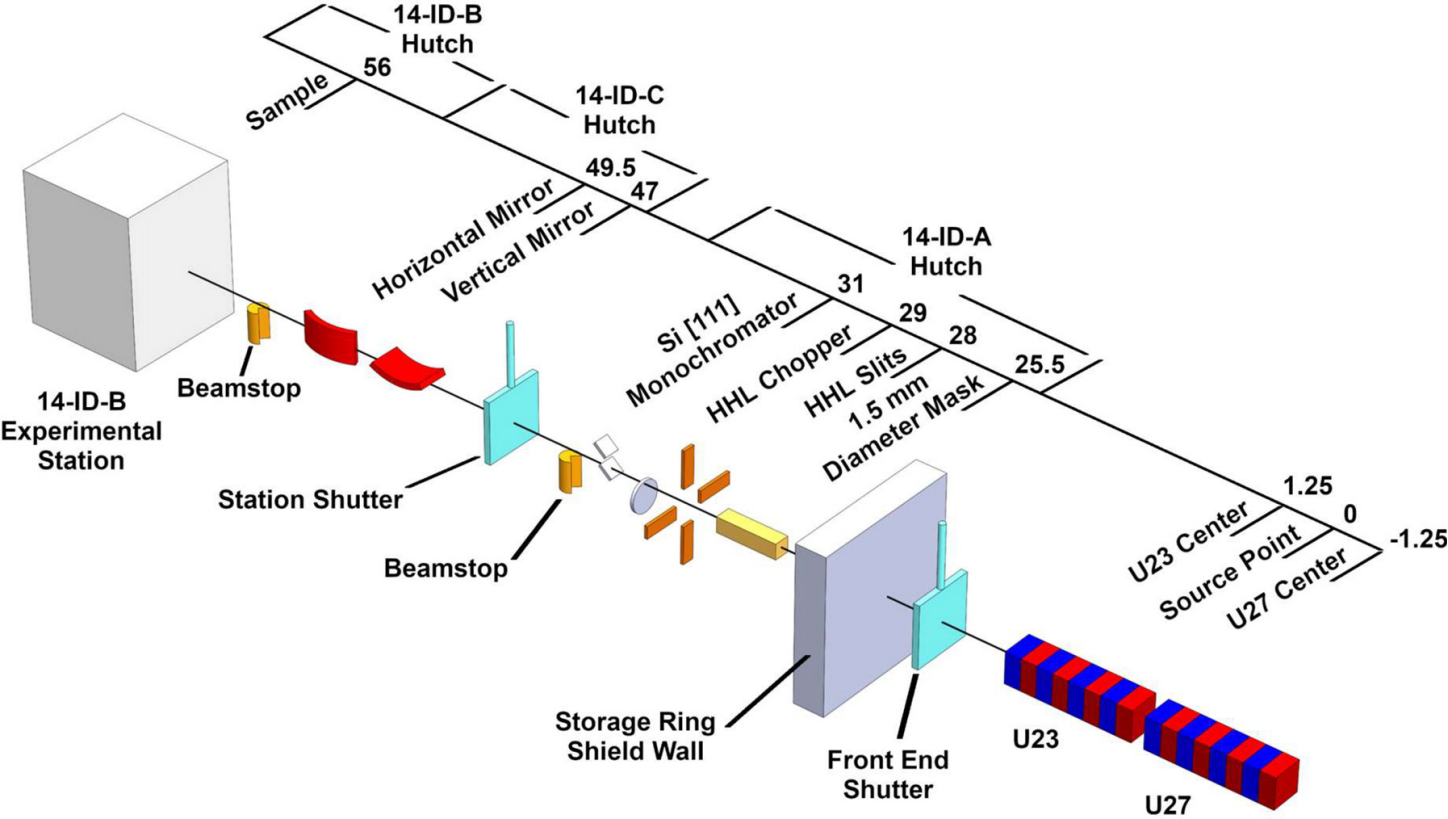
Current layout of the 14-ID beamline. Shown distance from the source point is in meters.

The unique beamline design of 14-ID takes advantage of the pulsed structure of the APS x-ray beam to deliver high-flux polychromatic single 100 ps x-ray pulses to the sample. This, in combination with highly demagnifying x-ray optics and choppers, produces high flux densities which are needed for all time-resolved experiments. Figure 1 shows the current layout of the beamline.

### Undulators

Time-resolved experiments are typically flux-starved so it is necessary to maximize the x-ray intensity as much as possible. Energy tunability is also desirable for some situations. To cover both scenarios, two colinear undulators, U23 and U27, were installed. U23 was optimized to deliver max flux at 12 keV and U27 can operate from 6.8 to 19 keV. Intensity is highest at 12 keV where most experiments are performed. With the ongoing upgrade of the APS, different undulators will be installed. Two new U21 undulators will still maximize the flux at 12 keV when both are used but can be tuned between 8.5 and 15 keV. The flux in a single 100 ps x-ray pulse at the sample position is currently 8 × 10^9^ photons/pulse in 24-bunch mode and 3 × 10^10^ photons/pulse in hybrid mode of the APS storage ring.

### 14-ID-A hutch

The 14-ID-A hutch contains a mask, white beam slits (HHL slits), heat-load chopper (HHL Chopper), Kohzu monochromator and the station shutter for the downstream stations (Fig. 1). Even though most experiments today utilize the focused polychromatic beam, a Kohzu HLD-9BC double crystal monochromator with cryogenically cooled silicon (111) is available for standard monochromatic experiments. The monochromator can be tuned from 6.8 to 19 keV with a fixed 15 mm offset.

### X ray beam focusing

The 14-ID-C hutch (Fig. 1) houses a water-cooled Kirkpatrick–Baez (KB) mirror system (Oxford-Danfysik) which was initially designed to handle the full-power white beam, and to control, independently, the vertical and horizontal focus without changing the mirror angles. The mirror angles can be adjusted from 2 to 4 mrad but are typically fixed at 3.8 mrad to maximize the flux at 12 keV. The vertical mirror has rhodium and platinum stripes with ∼3 mm of bare silicon between the stripes. The horizontal mirror has rhodium and silicon stripes. The rhodium stripes are used for energies above 10 keV. At lower energies, the mirrors are translated to the silicon stripes to suppress the higher harmonics emitted from the undulator.

The focal spot size produced by these primary mirrors is ∼20 × 70 *μ*m^2^ (V × H) and is typically near the Jülich chopper located in the 14-ID-B end station in order to achieve single pulse isolation by the chopper. Slope errors as measured by metrology are 0.9 and 1.04 *μ*rad, respectively but the small vertical focus indicates that the slope error of the vertical mirror is closer to 0.2 *μ*rad. This is attributed to the x-ray beam footprint being approximately one half of the mirror length and the slope errors near the middle of the mirror are lower. The horizontal mirror focus is dominated by the current large divergence of the APS beam.

The final focusing is accomplished by a second KB mirror system (Instrument Design Technology Ltd.) which is located in the 14-ID hutch, downstream of the Jülich chopper. This secondary mirror system was added in 2013. Before that, no additional focusing was available so the final focus was typically 60 × 90 *μ*m^2^ (V × H) at the sample position. The secondary KB mirror system pushed the minimum beam size to ∼20 × 20 *μ*m^2^ (V × H) at the sample position while maintaining the full intensity of the beam. Each mirror is 240 mm long and both are contained within a helium environment to minimize air scatter and to protect the surfaces of the mirrors from oxidation. Each mirror has both rhodium and silicon stripes and, like the primary mirrors, are translated to either position depending on the working energy for the experiment.

### 14-ID-B end station and sample environment

Samples are typically mounted on an air-bearing based rotation stage from ALIO Industries. The rotation stage can rotate up to 720 °/s and has a small sphere of confusion (∼1 *μ*m). Final sample positioning is done with two piezoelectric linear stages that are mounted on the rotation stage and a large linear stage that aligns the entire assembly to the x-ray beam.

To align the samples to the x-ray beam and to perform beamline diagnostics, two network based cameras are mounted 90° apart. The high magnification camera is mounted at 30° below the x-ray beam and the wide field camera is mounted at 60° above. This orientation allows for quick alignment of the sample. Both cameras are mounted on a rigid pedestal which is firmly mounted to the table.

A brass collimator is also mounted on the pedestal. The tip of the collimator can be exchanged with different aperture sizes depending on the requirements of the experiment. The collimator can be aligned by a set of XYZ stages. It is also purged with helium to minimize air scatter. Some experiments require a different configuration of the cameras and collimator, so the pedestal was designed to be removed from the optical table as one unit. This allows for other equipment to be mounted such as the serial crystallography configuration. In this setup, on-axis sample visualization is implemented which is also used for on-axis laser illumination. To return to the standard configuration, the pedestal can easily be remounted and aligned without requiring an extensive amount of time.

All these components are mounted on two separate optical tables inside the 14-ID-B end station. The Jülich chopper, JJ slits and secondary KB mirror system are mounted on the upstream table. This table is rarely moved and, once all these components are aligned, is typically not used for any ancillary equipment to maintain beamline stability. The downstream table has the ALIO goniometer, pedestal, x-ray detector and laser optics. Additional user equipment is also mounted on this table. Once the downstream table is ready for the experiment, it can easily be optimized to the x-ray beam without having to adjust any of the upstream components on the beamline.

### X ray detectors

The high flux in the short 100 ps polychromatic x-ray pulses requires an integrating x-ray detector. Counting detectors such as the Dectris Pilatus or Eiger detectors do not have a high enough counting rate. We use Rayonix MX340-HS CCD detector. The large active area (340 × 340 mm^2^) and small pixel size (∼88 *μ*m in standard 2 × 2 binning mode) makes it well suited for both crystallography and solution scattering experiments. It can be hardware triggered and is frequently operated at 10 Hz for serial crystallography experiments. 100 Hz operation is possible but requires a higher binning mode. This detector works well for most experiments, but the framerate became limiting factor for some experiments. This is especially true for serial crystallography experiments where we want to take full advantage of the chopper system which can operate up to 1 kHz. For these experiments, a low-background, fast frame rate integrating detector is preferred so a 2.1M ePix10k detector from SLAC was recently acquired. The detector consists of 16 modules, each with 352 × 284 pixels. The pixels are 100 × 100 *μ*m^2^ with a 14 bit well depth. The active area is 165 × 165 mm^2^. The current frame rate is 250 Hz, but future firmware updates are expected to increase the data collection rate to 1 kHz.

### Network and computational resources

As data collection rates are increased and data are processed in near real time, we have updated the data processing computers, data storage array and network to account for these demands. This is especially needed for the new 2.1M ePix10k detector operating at 1 kHz since it could generate approximately 14 TB of data in 1 h. To handle this amount of data, a 40 Gbps network interconnects the detector, the data storage, and the data processing computers. A new 900 TB BeeGFS based high-performance storage array has been installed and tested at these data collection rate. Two 56 core data processing computers are available to process the data. Each computer has many software packages available for data processing. BioCARS also has access to the high-performance computing clusters at both the University of Chicago and Argonne National Laboratory. We maintain our own globus end point which allows users to easily transfer their data to any place in the world with a web interface. Long term data storage is available on the APS voyager system.

### X ray pulse isolation

**FIG. 2.**
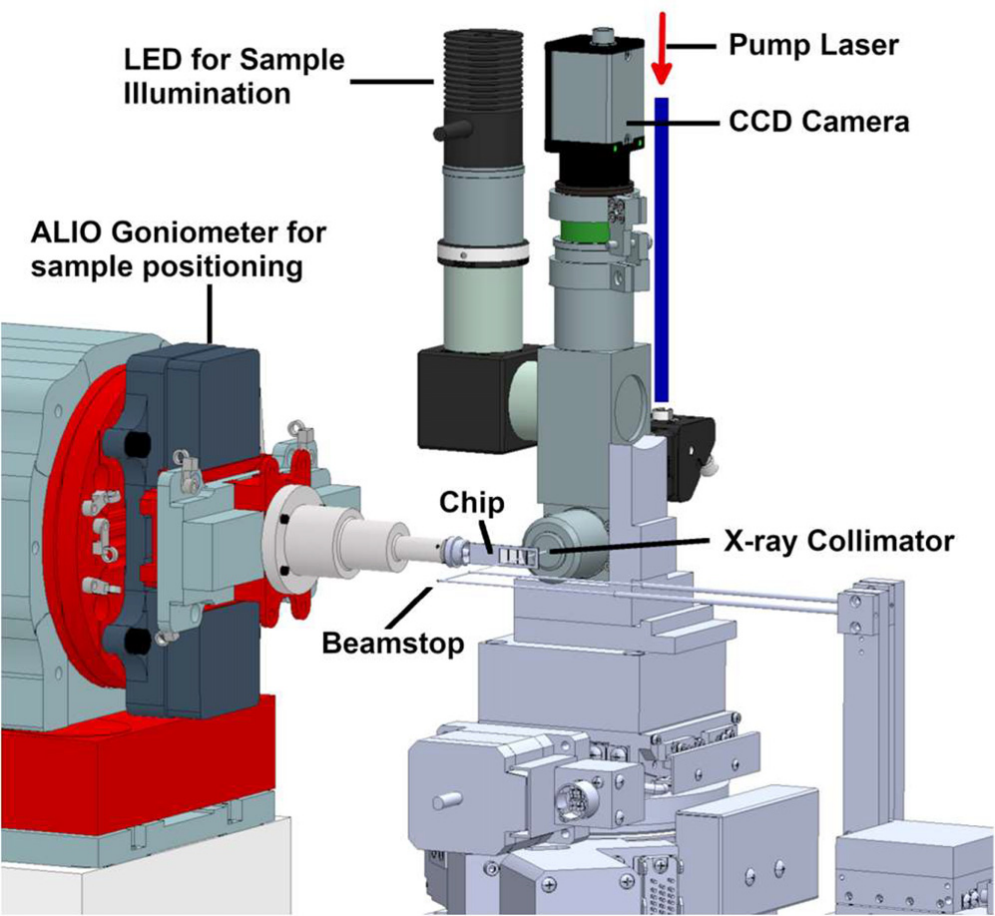
Serial crystallography setup at 14-ID experimental station.

X ray pulse isolation is achieved by using a series of choppers and shutters. This was described in detail in Graber *et al.*^67^ (Fig. 2) and we provide only a brief description here. The high heat-load chopper (HHLC) is the first chopper in the beamline (in the 14-ID-A hutch) and is operating continuously. It typically reduces the x-ray pulse duration to ∼20 *μ*s with a frequency of 82.3 Hz but other combinations are available as well. The ms-shutter, located in the 14-ID-B hutch, blocks the x-ray beam until it is triggered on demand by the timing FPGA system (see below). The ms-shutter has an opening time of 4–5 ms and each trigger allows one 20 *μ*s burst of x-rays to pass. The next major component is the ultra-fast Jülich chopper. This chopper is used for the final single x-ray pulse isolation. The chopper is synchronized with the APS and is mounted on two linear translation stages. Single pulse isolation can be achieved by two different methods: use of chopper tunnel to select a single x-ray pulse in 24-bunch mode of the APS storage ring or a tunnel-less mode. The tunnel-less mode allows either a single x-ray pulse or a series of consecutive x-ray pulses to be isolated by changing the vertical height of the chopper. This is especially useful when the 100 ps time-resolution of a single pulse is not required but additional intensity provided by multiple pulses is necessary.

### Beam diagnostics

In-vacuum four-blade x-ray slits (JJ Xray) are mounted on both the upstream and downstream sides of the Jülich chopper. This allows for cleaning up the tails of the beam. Beam diagnostics is incorporated in the rigid pedestal mentioned above, downstream of the chopper. Beam intensity is measured with a standard PIN diode coupled to an oscilloscope. This intensity is used to monitor the x-ray beam during an experiment. To monitor non-invasively the pump–probe delay during an experiment, a Bicron BC422Q scintillator coupled to a micro-channel plate photomultiplier is used, connected to a fast oscilloscope.

### Laser systems

BioCARS offers a variety of lasers for use by the scientific community. For experiments that require 100 ps time resolution, a tunable picosecond laser system is available. The laser system consists of a Spectra-Physics Spitfire Pro picosecond laser coupled to a TOPAS optical parametric amplifier. Tunable wavelength range is 250–2000 nm. To maintain intensity stability and to minimize positional drift during an experiment, the laser system must be kept thermally stable to within 0.1 °C. This is achieved by locating the laser system in a separate laser room, isolated from the x-ray hutch. The laser beam is transported by a series of mirrors from the laser room to the 14-ID-B hutch. The laser pulse length is ∼1 ps but can be stretched to ∼35 ps with an echelon. The ps laser can operate up to 1 kHz.

Experiments that do not require 100 ps time resolution may benefit from a longer laser pulse length so two different OPOTEK nanosecond lasers are available. An Opolette 355 HE Nd:YAG/optical parametric oscillator (OPO) laser system is used primarily for UV/Vis experiments and an Opolette 532 LD Nd:YAG/OPO is used for IR studies. The tunable range between the two lasers is from 210 to 2200 nm. The small footprint allows both lasers to be permanently mounted on the laser beam conditioning optics support above the experimental table in 14-ID-B end station. The OPOTEK lasers operate at a max frequency of 20 Hz. Several CW lasers and laser diodes are also available at various wavelengths. Many of these can be used in a pulsed mode, with pulse durations in the millisecond range.

### Synchronization

Time-resolved experiments require precise timing of all components down to the ps level. To achieve this, a Xilinx Virtex-II Pro Field Programmable Gate Array (FPGA) with an embedded Power PC processor is used to monitor two timing signals (352 MHz and 271 kHz) from the APS and then trigger the required choppers, lasers, shutters, stages, oscilloscopes, and detectors. The FPGA was developed by the Philip Anfinrud group (NIH/NIDDK) and is used for all experiments at BioCARS. In addition, an external GigaBaudics, 3 GHz Programmable Delay Line, model PADL3-10-11, is used to generate pump-probe delays with 10 ps resolution. One issue with this FPGA implemented in 2008 is how fast it can change the time delay between the laser and the x-ray between x-ray images. This is currently done remotely through the network, and it takes ∼1 s to update. A new version of the FPGA is being implemented that calculates all time delays ahead of time and then are downloaded to the FPGA. This will remove the ∼1 s delay and the data collection can proceed at a much higher frame rate. To improve the frame rate even further, devices which are controlled through software will be hardware triggered.

Experiment setup and data collection are managed through Lauecollect, a custom Python-based software environment developed at BioCARS. Lauecollect controls and automates the data collection process through a graphical user interface (GUI) that permits users to input all experiment-specific settings such as pulse protocols for reaction initiation and diffraction, angular settings, exposure times and communicates with the FPGA timing system, motor controllers, oscilloscopes, and detectors.

### Data analysis

The new experiment designs and data collection at BioCARS can be technically challenging, but remarkably, the analysis and interpretation of the data often represent the rate limiting steps in overall progress. The reasons are quite clear. The goal in dynamics experiments is typically to induce subtle perturbations to the ground state of molecules to expose their natural (rather than non-physiological) degrees of freedom. The subtlety of perturbations, however, raises serious challenges for practical data analysis. We expect only slight changes in x-ray scattering intensities, and these small signals must be robustly separated from the many sources of random and systematic noise that plague experiments. In addition, work at BioCARS is carried out with polychromatic x-rays to maximize photon flux and optimize data collection efficiency, requiring different approaches for data reduction and analysis than traditional experiments at monochromatic sources. To address these issues, BioCARS has worked with core users over the past five years to develop specialized algorithms (and associated software) for Laue data processing and robust signal detection in both crystal- and solution-based x-ray scattering experiments.^69–71^ These advancements help build the foundation for a more comprehensive computational infrastructure for the future of macromolecular dynamics.

## TIME-RESOLVED LAUE CRYSTALLOGRAPHY AT BioCARS

Complete infrastructure for standard, single crystal time-resolved experiments with samples that undergo reversible reactions has been available and used at BioCARS for over 20 years, initially with ns time resolution and since upgrade in 2007–2008 with 100 ps time resolution. The traditional method of reaction initiation has been use of short laser pulses. In recent years, we focused our activities in addressing two challenging areas of this field: studies of irreversible reactions and expanding the methods of reaction initiation beyond light. To address the challenge of irreversible reactions while minimizing sample consumption, we implemented time-resolved serial Laue micro-crystallography using fixed targets and crystal injectors. To address the challenge of providing a more general method to initiate reactions and dynamic responses in biomolecules, we implemented technologies for electric field and temperature jump crystallography. We describe these experiments briefly below.

### Time-resolved serial Laue micro-crystallography (TR-SMX) at BioCARS

Serial micro-crystallography (SMX) was originally developed due to necessity for use at the new XFEL sources. Diffraction-before-destruction at these high brilliance sources required high-throughput sample delivery methods where fresh crystals are supplied continuously and each crystal at near-room temperature is illuminated by an ultra-short x-ray pulse only once. For synchrotron application, serial micro-crystallography mitigates the need to obtain large, well-ordered single crystals and reduces issues of radiation damage at ambient temperatures. For time-resolved crystallography as conducted at BioCARS, serial crystallography approach provides an efficient and general path for time-resolved studies of irreversible reactions. For such reactions, a fresh crystal is necessary for each pump-probe cycle in a time-resolved experiment. For example, understanding and manipulating enzymatic reactions starts with measuring the motions that define the reaction coordinate. However, many enzymatic reactions are irreversible, single pass reactions (substrate binds to the enzyme, is turned over, and product is released). Studies both at XFEL sites and at BioCARS^37,40,42,72–74^ validate the argument that serial crystallography, combined with reaction initiation based on diffusion or photo-activation of caged-compounds, represents a general-use methodology to probe the dynamical basis for protein function—a missing tool in structural biology.

What uniquely suits BioCARS for TR-SMX? First, synchrotron sources provide excellent brilliance and time-resolution, with an exceptional x-ray beam stability in temporal, spatial and spectral dimensions. At the 14-ID beamline, with polychromatic beam capability, we provide ∼3 × 10^10^ photons in a single 100 ps x-ray pulse in the hybrid mode of the APS storage ring, focused to 20 × 20 *μ*m^2^. This exceptionally high flux limits the time resolution for data collection only by the 100 ps duration of the synchrotron pulse.

A second critical advantage at BioCARS is efficiency in data collection. In traditional monochromatic crystallography, integrated intensities of reflections are obtained by rotation of the crystal during x-ray exposure; integration is obtained over angle. However, the nature of serial crystallography experiments using any x-ray source and method is that crystals generally cannot be rotated during x-ray exposures. When these experiments are conducted using monochromatic synchrotron beamlines (ΔE/E of ∼10^−4^) or “quasi-monochromatic” XFELs (ΔE/E of ∼5 × 10^−3^), all recorded reflections are “partials”—that is, the recorded intensity of a reflection only represents a portion of the full structure factor amplitude. One possible solution to this problem is to collect many diffraction images with randomly oriented micro-crystals, with the simple idea that summation of these partials will converge to quantities proportional to the full intensities as the number of indexed images becomes large.^75,76^ Typically, 1000–100 000 such indexed “still” images are necessary to assemble a complete dataset.^77–84^ BioCARS offers a fundamentally different approach to the partiality problem. With the ∼3%–5% bandpass of the first harmonic of the BioCARS undulator source, each diffraction image recorded from a stationary crystal is a Laue pattern in which integration of structure factor amplitudes is achieved over the x-ray energy rather than over the crystal rotation angle.^85^ Less than 100 diffraction images can be sufficient to acquire a complete Laue dataset using a single crystal; the exact number depends on the crystal space group and the extent of redundancy required. With a stream of randomly oriented micro crystals in a serial crystallography experiment at BioCARS, sufficient number of diffraction patterns is on the order of a few hundred.^86,87^

### Conducting time-resolved SMX experiments

Our standard crystallography setup for work with larger, MiTeGen-loop mounted crystals has two off-axis sample viewing cameras. However, this setup is not well suited for serial micro-crystallography where crystals are presented on chips, tape drives or crystal injectors. As mentioned earlier, the standard configuration can be quickly swapped out with the new serial crystallography setup. This new setup developed in collaboration with Alke means group (CFEL/DESY, Germany) (Fig. 2) has an on-axis view of the sample at high magnification which uses compact standard microscope objectives. The same viewing optics can be also used to deliver on-axis laser illumination to the sample for time-resolved experiments. The full range of wavelengths cannot be covered with one configuration of optics, lenses, and beam splitters, so two identical setups are necessary and available that can be easily swapped out and realigned to the x-ray beam. One covers the UV/VIS range of laser wavelengths and the other works in the near IR range (out to 1500 nm). To minimize background, which is of particular importance when using microcrystals, an x-ray collimator is integrated with microscope objective to provide a clean x-ray beam to the sample.^86^ In addition, a small, collimating beam stop is used close to the sample to capture most of the air scatter from around the sample position. The air scatter is reduced even further by using a helium environment around the sample. For these experiments, we can also provide a temperature/humidity chamber that works from −10 °C to 60 °C with an adjustable relative humidity between 80% and 100%.

BioCARS offers several sample delivery methods for serial crystallography. The most common approach uses fixed target devices, which involves placing the sample on a thin support and then rastering the sample through the x-ray beam. A variety of materials could be used for the support, but silicon chips and Mylar are the most common. The Meents group^88^ developed a silicon chip version which is now commercially available and is available at BioCARS. The ALEX mesh-holder design by the SBC group at the APS^89^ is also popular and was successfully used to collect time-resolved serial crystallography data at BioCARS.^42^ These chips enable successful data collection with 10–40 *μ*m crystals and single 100 ps x-ray exposures, using as few as 50–200 images for complete datasets.^86^ To optimally use the beamtime, we continuously implement ways to increase the data collection rate. In this case, new piezoelectric stages currently being implemented (SmarAct SLC-1720) will permit data collection rate of up to ∼40 Hz.

BioCARS also offers to users a high-viscosity extrusion injector (HVE).^80^ This injector can be used with lipid cubic phase (LCP), grease matrix or other viscous crystal carrier materials, at much lower injection velocities of 0.1–1 mm/s than XFEL jets. Importantly, this crystal injector is compatible with light induced reaction initiation.^90^ Our users successfully demonstrated Laue experiments using the HVE injector at BioCARS. Laue data were collected with 15 × 10 × 5 *μ*m^3^ proteinase K crystals in LCP matrix and exposures of four consecutive x-ray pulses in 24-bunch mode of the APS.^87^ The data resulted in an excellent electron density map to 1.8 Å derived from 132 merged diffraction images. Data were also collected with significantly smaller 5 × 5 × 2 *μ*m^3^ crystals of A2A adenosine receptor (A2AAR) to 4.2 Å resolution.

Fixed target chip devices and crystal injectors are well suited for light-initiation of structural changes, such as in experiments with naturally photosensitive proteins or with enzymatic reactions in which light-activated caged compounds are used, or for temperature jump experiments. Another method of reaction initiation, mix-and-inject, involves diffusion of substrates into crystals of enzymes.^37,40,91^ BioCARS provides two different methods to implement this approach. In one option, we use a tape drive to enable reaction triggering by a mix-and-diffuse approach in which the mixture is continuously deposited onto the tape by using two syringes. A second option is to use the mix-and-inject method in which a microfluidic device permits mixing and rapid diffusion of substrates into protein microcrystals. BioCARS has collaborated with Lois Pollack's group (Cornell University) to implement mix-and-inject experiments at BioCARS. This group developed microfluidic mixers and injectors for use at XFELs where first time-resolved mix-and-inject crystallography experiments were conducted.^40,73,92,93^ They recently adapted this technology for use at synchrotrons (manuscript in preparation) and we plan to implement and offer this technology to all BioCARS users.

### The electric field and T-jump crystallography at BioCARS

As we discussed, direct and fastest stimulation of the reaction coordinate with the goal of visualizing the resulting motions is accomplished by using short light pulses, both for photo-activation of native chromophores or caged-compounds in the absence of native chromophores. Another, slower method of reaction initiation involves substrate diffusion.^91^ However, a different (and more general) approach to macromolecular dynamics is to nonspecifically and globally induce dynamics to elucidate all possible internal motions. The inspiration of this approach is that like any machine of conventional familiarity, protein function relies on programmed, low-dimensional collective motions that define the reaction coordinate, and that a global but subtle perturbation analysis will expose these motions effectively.^94^ This goal is the inspiration for the electric-field stimulated time-resolved x-ray diffraction, EFX.^94^

In the EFX approach, the idea is to initiate conformational changes within protein crystals with an external electric field (the “pump”) and then to readout the structural response at any time delay using timed, x-ray pulses (the “probe”). This method can subtly excite and record motions throughout protein structures in the sub-nanosecond to microsecond scale, and evidence suggests that these motions are strongly related to conserved biological functions. EFX greatly expands the generality and scope of time-resolved studies to any protein for which crystals of suitable quality can be grown. What is the basic principle of EFX? Like all macromolecules, proteins have many charged species—fixed, dipole, or higher-order moments—distributed throughout their tertiary structure, and basic physics argues the application of an external electric field will exert forces on these charges that will induce motions and globally perturb their structures and reactions. If the electric field is applied in conjunction with fast, timed x-ray diffraction in protein crystals, it is possible to observe all induced motions with high spatial and temporal detail. Since its introduction, substantial progress has been made at BioCARS to make EFX a relatively turnkey method capable of execution by the typical user groups at this facility. Indeed, advances in electrode fabrication, crystal mounting, dedicated instrumentation for delivery of electric field pulses, and data collection strategies make it so even a single user can collect complete EFX datasets.

From a data-collection perspective, the temperature jump method is merely a special case of laser induced reaction; the idea is to use infrared laser pulses to rapidly heat the solvent within a protein crystal, followed by thermal diffusion to induce motions of protein atoms and timed x-ray diffraction to readout the response.^95^ As with EFX, this approach causes subtle, global perturbations to macromolecular structures, with the goal of visualizing full range of motions made possible by the pattern of internal constraints.

### Conducting time-resolved EFX experiments

**FIG. 3.**
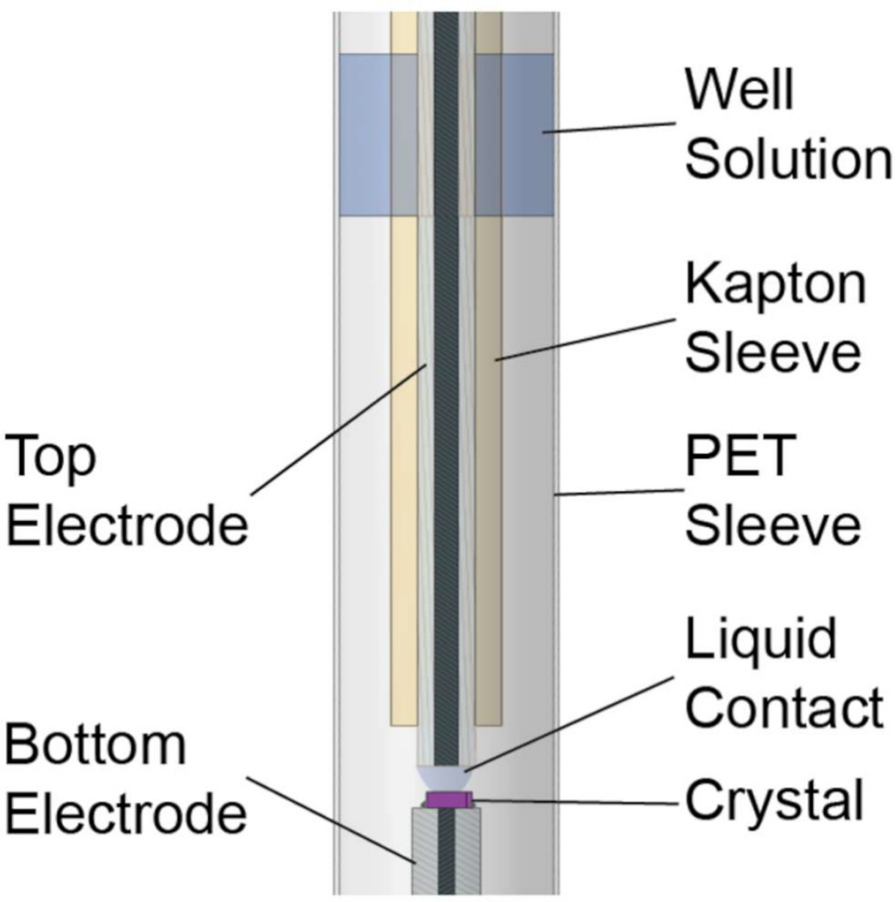
Improved sample cell design for EFX experiments.

The original EFX sample/electrode apparatus was described in Hekstra *et al.* A more recent and improved design minimizes fluctuations in ambient humidity during assembly of the final EFX configuration and eliminates mismatches between the solutions used for bottom and top electrodes (Fig. 3). Single crystals are physically fixed to a grounded bottom electrode using a dielectric glue that forces the electric current to flow through the body of the crystal (rather than around the edges) and stabilized within a mylar sleeve and a drop of mother liquor to maintain crystal humidity. A top electrode, connected to the field generator, is then positioned using an XYZ micromanipulator, driven through the mother liquor to acquire a drop of solution at the tip and then made to contact the crystal through the liquid interface. This geometry enables electric field pulses in the range of 1 MV/cm to be applied across the crystal (the pump), with precisely timed x-ray pulses to readout the structural response (the probe).

To apply the electric field, BioCARS uses a custom bipolar pulse generator that allows us to programmatically apply and invert the sign of the electric field with zero delay during data collection. The ability to collect data with the electric field applied in both directions enables a comparative analysis between the two to separate subtle but biologically relevant electric field-induced motions from those induced by unwanted processes such as heating or radiation damage. Voltage up to 10 kV can be applied to the sample but is typically in the range of 4–8 kV. Every sample requires some testing to determine the proper voltage. Too low of a voltage and no atomic motion will be detected. Too high of a voltage can cause the diffraction spots to be elongated which can be more difficult to process. Data are collected using a standard single time-resolved Laue data collection. Both voltage-ON and voltage-OFF data are collected at each angular setting of the crystal, the sample is then rotated 1°–3°, and time delays are repeated until all required data has been collected. The full angular range required depends on the crystallographic symmetry of the sample.

### Examples of time-resolved crystallography experiments

Time-resolved serial crystallography was recently used to reveal the binding and cleavage of moxalactam antibiotic by L1 metallo-β-lactamase (MBL) from *Stenotrophomonas maltophilia.*^42^ MBLs are enzymes produced within the bacteria that breakdown β-lactam antibiotics, making these microorganisms multi-drug resistant since β-lactam-based antibiotics account for ∼65% of all antibiotics currently used. Examples of these antibiotics include penicillins, cephalosporins, monobactams, and carbapenems. Moxalactam is a broad-spectrum, third-generation cephalosporin antibiotic. This particular MBL belongs to a B3 subclass that can breakdown almost every antibiotic containing β-lactam ring. A large, open active site of the MBL contains two Zn^2+^ ions, essential for anchoring the β-lactam ring in place and playing the key role in the substrate binding and catalysis.

For this experiment, 20 × 20 × 100 *μ*m^3^ crystals were loaded on ALEX chip mesh and chip was scanned through the x-ray beam using miniature fast translation stages.^89^ The reaction was initiated by photo-releasing of the Zn^2+^ ions from UV-labile zinc-photocages soaked into the crystals. At each sample position during a chip scan, Zn^2+^ ions were released using 7 ns laser pulses at 347 nm. Diffraction data were collected using either 24 or 48 consecutive x-ray pulse exposures (pulse train durations of 3.7 *μ*s or 7.4 *μ*s, respectively). Ten snapshots (from 20 ms to 4 s) at 2.2 Å resolution revealed metal ions binding to the active site, followed by binding of moxalactam. The intact β-lactam ring is observed for 100 ms after photolysis, as the electron density for an unhydrolyzed moxalactam increases from 20 to 100 ms and its conformation adjusts. The cleavage is detected at 150 ms. The reaction product then adjusts its conformation to finally reach a steady state corresponding to the relaxed state of the enzyme at 2 s.

One of the major strengths of BioCARS is flexibility of the sample environment with ability to accommodate user-developed experimental setups. One successful example is the setup developed by Yang/Ren group from University of Illinois Chicago. They developed a stand-alone platform for automated *in situ* serial crystallography at room temperature and demonstrated successful use of this platform with Laue diffraction at BioCARS.^96–98^ The platform eliminates sample manipulation of the often-fragile crystals. It is also compatible with light-induced time-resolved studies as demonstrated by using the photosensory core module of a bacteriophytochrome to obtain a difference electron density map which clearly showed light-induced structural changes after 500 ms illumination by far-red light at 785 nm.^96^

Recent use of the EFX method at BioCARS involved resolving conformational changes in E. coli dihydrofolate reductase (ecDHFR), a model system for the role of protein dynamics in catalysis.^99^ To elucidate coupled conformational changes in this enzyme, authors used x-ray crystallography, while applying various perturbation methods: ligand-, temperature-, and electric-field-based, as well as molecular dynamics simulations. While the multi-temperature diffraction experiments resolved a global hinge motion that constricts the active site cleft and identified networks of correlated motions, EFX experiments conducted at BioCARS at room temperature revealed electric-field-dependent constriction of the active site cleft. At each orientation of a single crystal, three time points were collected: an OFF reference timepoint (no high-voltage pulse), a 200 ns timepoint during a 3.5 kV pulse, and a 200 ns timepoint during a −3.5 kV pulse. To collect a complete dataset, crystal was rotated and same timepoints were repeated at each angular setting of the crystal. Conformational rearrangements in functionally important regions of the protein induced in these experiments are similar and consistent with those observed in the multi-temperature experiment. Taken together, results from this multi-method approach support a common mechanism in which the global hinge motion is coupled to local rearrangements throughout the enzyme on the nanosecond timescale.

### New developments in Laue data processing

The Laue data processing program Precognition (Renz Research), used for many years at BioCARS was originally written for processing data from single crystals. However, for processing of serial data where each image is indexed independently, improvements were necessary to automate and speed up data processing. We developed a Python GUI for this purpose. The interface facilitates hit finding, with custom criteria for defining hits. Images that are considered hits are then independently processed by Precognition in parallel to speed up indexing, geometry refinement, and integration. We also implemented features for automated rejection of images reported indexed but which contain multiple diffraction patterns resulting from several crystals exposed at the same time or images that are actually mis-indexed. Another important issue for serial crystallography is resolving indexing ambiguity which exists for some space groups. For this purpose, we currently use an independent program,^100^ but the plan is to include this step into future processing pipelines for serial Laue data. Other areas of Laue data processing that require improvements, especially with micro-crystals data, include indexing of very weak and sparse diffraction images, as well as more accurate integration of streaky diffraction patterns.

Another recent development in Laue data processing involves a newly developed open-source program Careless for scaling and merging of crystallographic data, including Laue data.^69^ The program was developed by Doeke Hekstra group at Harvard University and has been successfully tested with recent time-resolved serial crystallography data from BioCARS, in addition to some standard BioCARS time-resolved data already used for benchmarking the program. The program for now uses as input integrated intensities from program Precognition. However, development of a new and complete Laue data processing tool, Laue-DIALS is under way. A first version of the software, for now suitable for indexing of single crystal Laue data, is available on GitHub (https://github.com/rs-station/laue-dials) as an extension to DIALS code, a widely used open-source software for the analysis of diffraction data.^101^

We have also recently used the CrystFEL software package^102^ to process BioCARS serial time-resolved Laue data. This program was originally developed for processing of quasi-monochromatic XFEL serial crystallography data but it has been also already used for processing of wider-bandpass (2.2%–2.5%) Laue data, both from synchrotron and from XFEL sources^103,104^ For indexing of Laue data it was necessary to develop a new program, *pinkIndexer.*^70^ To evaluate *pinkIndexer* using real Laue serial crystallography data, authors used the data from Meents *et al.* measured at BioCARS. In addition to Laue-specific indexing (*pinkIndexer*) which is implemented in some versions of CrystFEL, several additional steps are necessary for Laue data processing before merging of data with CrystFEL. These include unit cell scaling and scaling of integrated intensities by taking into account measured x-ray spectrum. These steps have been implemented by Alexandra Tolstikova outside of CrystFEL as Python scripts. Complete CrystFEL-based Laue processing pipeline has been described by Tolstikova in chap. 7 and depicted in Fig. 7.12 of Tolstikova 2020 Ph.D. thesis.^105^

## TIME-RESOLVED SOLUTION SCATTERING AT BioCARS

Small angle x-ray solution scattering (SAXS) provides a plenitude of information about biomolecules in their native solution state, such as size, molecular weight, flexibility, structural homogeneity, fold, and shape.^47,106,107^ This approach is complementary to x-ray crystallography, providing a suite of tools for detecting flexibility, structural inhomogeneity, and solvent interactions in the solution state. These measurements are essential for understanding biomolecule function. At BioCARS, we extend the solution scattering to collect temporal dynamics, enabling the scientific community to go beyond static descriptions and characterize the changes in structure over time that underlie function.

The initial time-resolved solution scattering (TRXSS) experiments at BioCARS were conducted with photo-sensitive protein systems, such as myoglobin,^68,108^ hemoglobin,^109^ and photoactive yellow protein,^110^ and this work provided important insight into structural changes induced by ligand dissociation, allosteric transitions, and signal transduction. These successes justified the expansion of solution scattering technologies to encompass more general methods of reaction initiation, including temperature-jump (T-jump) and use of caged compounds such as protons and cations. Examples of such experiments conducted at BioCARS include de-caging protons by a short UV-pulse for inducing PH-jump^111^ and conducting temperature jump studies of protein folding,^112,113^ ligand binding,^114^ enzymatic specificity^113^ and protein oligomerization,^115^ protein-solvent interactions,^116^ dynamics of ensemble populations, etc.^117,118^

### Conducting time-resolved solution scattering experiments

In typical time-resolved solution scattering configuration at 14-ID-B, polychromatic x-ray beam is used at peak energy 12 keV and energy bandpass of ∼2.5%. In this configuration, a single 100 ps polychromatic x-ray pulse delivers 5 × 10^9^ photons and is focused to a spot of ∼30 × 30 *μ*m^2^ (V × H).

A typical pump-probe TRXSS experiment at BioCARS involves reaction initiation in the sample by a short laser pulse (pump), then using a single 100 ps x-ray pulse or a train of single pulses to probe the reaction after a specific time delay. The sample is then refreshed, typically by scanning the sample cell to a new spot, or by flowing the sample in a flow-cell. The procedure is repeated until appropriate signal-to-noise is achieved before the detector readout. Typically, with 100 ps x-ray exposures, necessary for sub-500 ns time resolution, several thousand pump-probe cycles are needed to collect one scattering image with sufficient signal-to-noise ratio. For a comprehensive time-resolved experiment, it is usually necessary to collect ∼10 time delays per decade in time. Because structural changes are typically small, they often result in less than 1% change of the scattering intensity. We, therefore, utilize difference technique, so for each laser-ON image we also collect laser-OFF image and use ON and OFF images to obtain difference signal. The difference technique allows us to minimize effects of slow change in experimental conditions and to increase signal-to noise of experimental data. The maximum frequency of data collection (pump-probe-refresh) is determined by the laser repetition rate.

To minimize parasitic scattering and detect small time-resolved signals, we use helium-purged cones, which are mounted at the face of the detector The cones are designed to cover minimum scattering q-region from 0.008 1/Å (540 mm length cone) to 0.025 1/Å (184 mm length cone), and maximum scattering vector of 1.8–5.5 1/Å, respectively. Overall, study of biomolecules with up to ∼300 Å in maximum dimension is possible, while also reaching the higher, wide-angle scattering (WAXS) q-range. Simultaneous SAXS/WAXS capability, coupled with high-flux polychromatic x-ray beam delivered in pulses as short as 100 ps to microsecond pulses, has enabled a variety of research projects which study biomolecules on global, hierarchical scale.

BioCARS offers experimental setups for several types of TRXSS experiments: (1) studies of photoinduced reversible reactions, (2) T-jump studies, and (3) studies of irreversible reactions with the emphasis on sample minimization.

For studies of reversible reactions, such as those typical for naturally photosensitive proteins, the sample can be recycled if it is not damaged by x-rays or intense laser pulses. The sample is loaded in a syringe or a conical tube, then delivered to a standard 0.5 mm diameter quartz capillary sample cell. The cell support is mounted on ALIO diffractometer and can be translated through the x-ray beam. Sample can be also flown through the capillary, then the capillary cell slowly moved, to avoid multiple x-ray exposures of the same spot on the capillary and to minimize sample radiation damage. The minimal quantity of the sample for a complete time-resolved dataset in this case is ∼250 *μ*l. This method can also be used for irreversible reactions, but sample consumption can be prohibitively high.

In T-jump studies ns laser pulses at 1443 nm are used to excite vibrational modes of water molecules in the buffer, then a fast transfer of the vibrational energy to the protein occurs. In collaboration with BioCARS users we pioneered T-jump solution scattering, with the first work^119^ reporting studies of protein–protein dissociation and reassociation dynamics in insulin, a model system for protein oligomerization. Since this first experiment, T-jump reaction initiation became one of the most popular methods among BioCARS users. One of T-jump studies revealed distinct motions in a protein folding chaperone cyclophilin A(cyt-A).^113^ Another T-jump study investigated intermediates of a HIV-1 Envelope (Env) glycoprotein construct on a microsecond timescale at different temperatures.^114^ In most cases, T-jumps are used to study reversible reactions where sample can be reused unless it is damaged by x-rays.

**FIG. 4.**
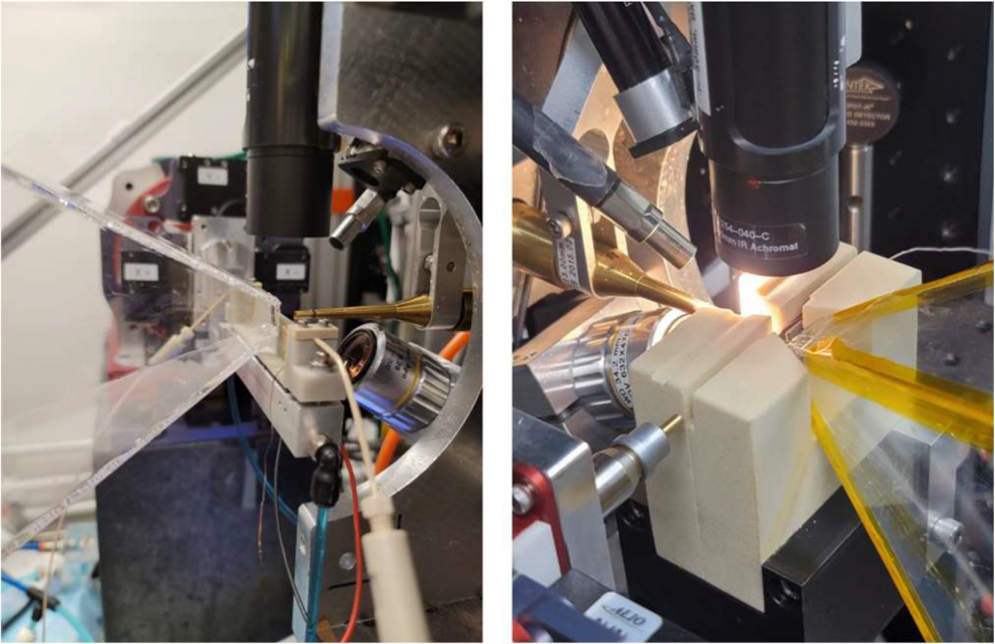
BioCARS solution scattering cells with temperature control. Left: AlN block cell; right: oven style cell.

The laser light in the T-jump experiments is typically delivered orthogonally to the x-ray beam and focused to ∼100 × 500 *μ*m^2^ spot size with power density of ∼50 mJ/mm^2^ to achieve temperature jump of 8 °C–12 °C. These classes of experiment require fine tuning of the initial sample temperature for the temperature jump to enable required kinetic process, or to initiate T-jumps from a series of temperatures to determine entropy and enthalpy of the process.^113^ In collaboration with Lin Chen's group (Northwestern University) we developed a heating cell, which is compatible with BioCARS sample environment. The cell consists of a quartz capillary, attached to an Aluminum Nitride (AIN) block, which is heated with Peltier elements (Fig. 4, left). This cell is available to all BioCARS users. The cell works in 15 °C–70 °C temperature range and has stability of 0.25 °C (in lower temperature range) to ∼1 °C (in higher temperature range). The maximum temperature jump using this cell is limited to ∼12 °C by the Aluminum Nitride damage threshold. For the experiments which require improved temperature stability, or higher temperature jump, in collaboration with Philip Anfinrud's group (NIH/NIDDK) we developed oven-style heater, where capillary is not in direct contact with the heater material. The cell is depicted in Fig. 4, right. The capillary with sample is placed between the aluminum plates of the heater, which are heated by Peltier elements. In this heater the temperature stability is below 0.1 °C in the temperature range from 4 °C to 80 °C and temperature jump that can be achieved is limited only by the ns laser power density that can be delivered to the sample.

**FIG. 5.**
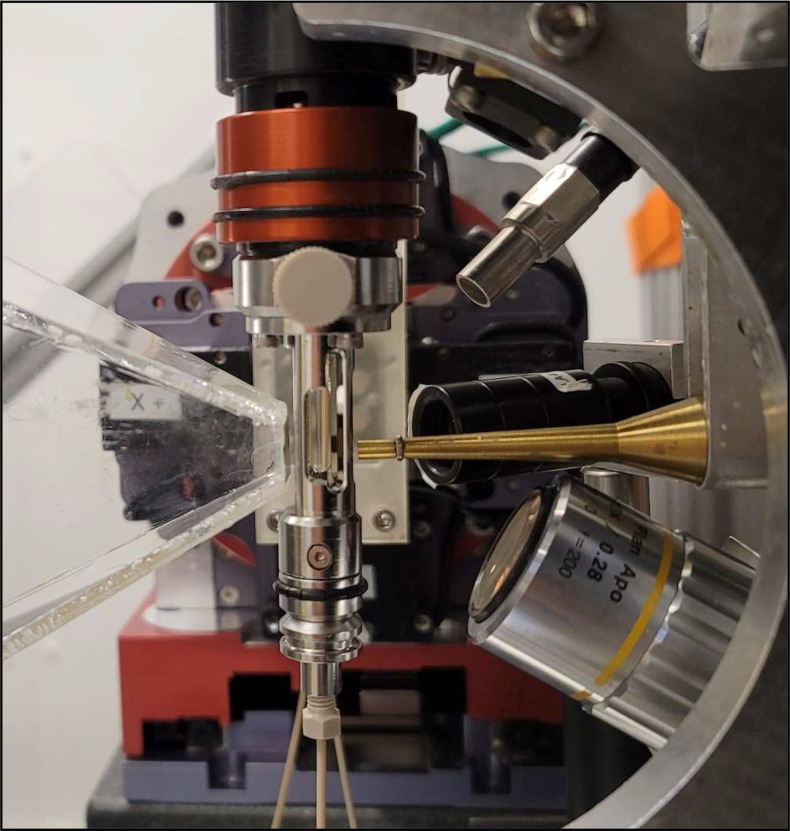
BioCARS co-flow cell for solution scattering studies of irreversible reactions. Reproduced with permission from Kosheleva *et al.*, “Sample-minimizing co-flow cell for time-resolved pump–probe X-ray solution scattering,” J. Synchrotron Radiat. **30**, 490–499 (2023). Copyright 2023 International Union of Crystallography.^120^

Many reactions of interest are irreversible. For those samples it is essential to minimize sample consumption since samples cannot be reused. In the standard capillary flow experiment in the laminar flow regime, the speed of the liquid at the capillary wall is approaching zero. In order to refresh most of the sample in the capillary cell and to minimize radiation damage of the sample in the capillary wall proximity, a large sample volume is required. Consumption in this case may reach up to a hundred ml during one time-resolved experiment with 100 ps time resolution. In collaboration with Kusel Design (Australia) we developed sample-minimizing sheath co-flow cell,^120^ where sample is injected in the buffer stream, so sample is not in contact with the capillary wall and, therefore, is not damaged by the x-ray pulses. The cell can be mounted either horizontally or vertically to accept laser light in orthogonal or collinear geometry (Fig. 5). The sample consumption in this cell is up to ten times smaller than in the typical capillary-scanned TRXSS experiment.

BioCARS data collection software Lauecollect, developed originally for time-resolved crystallography, has been expanded to include TRXSS data collection as well. Following the parameters setup, data collection is executed automatically without user intervention, including translation of the capillary cell and replenishing the sample. The software also monitors the status of the APS x-ray shutters and data collection is paused if shutters close to prevent sample loss.

For processing and preliminary analysis of experimental TRXSS data on-the-fly, BioCARS developed in-house software SAXS21D. Software uses information from the data collection log file to parse and process the data images. The software is GUI-based, written in Python. Recently, we upgraded our in-house software modules for image integration and correction with open-source modules pyFAI, Fabio.^121,122^ These modules allow wide range of flexibility in image processing. On the data processing server, it takes ∼0.5 s to process one image, which is adequate for all types of TRXSS experiments. Processed data are saved in the text format, compatible with ATSAS.^123^ In addition, data are saved hierarchically in hdf5 format, which users can view or parse by any compatible program. The SAXS21D software provides essential on-the-fly feedback to users. The feedback allows users to observe difference scattering signals as a function of time delay during data collection and to detect problems during the experiments. For example, one can detect promptly lack of the difference signal, decreased amplitude of the time-resolved signal, bubble formation due to intense x-ray or laser pulses, or sample deterioration. The SAXS21D software also includes singular value decomposition (SVD) analysis, which helps the user to assess the number of intermediates present in the collected time series and determine approximate transition times between intermediates. The program also allows merging different datasets and prepares the data for downloading for further data analysis.

### Examples of time-resolved solution scattering experiments

An example of a conventional TRXSS experimental study of a photosensitive protein is a study of a light-induced protein structural dynamics in bacteriophytochrome, BphP.^118^ The study provides mechanistic insights into how modular signaling protein BphP transmits structural signals over long distances and regulates its downstream biological response upon a red-light illumination. BphPs are photoreceptors that regulate biological mechanisms through red light–absorbing (Pr)–to–far-red light–absorbing (Pfr) reversible photoconversion, but structural dynamics of Pfr-to-Pr photoconversion in solution is not well understood. Therefore, a time-resolved experiment was conducted at BioCARS to elucidate details of photoinduced structural dynamics in PaBphP, a bathy BphP from *Pseudomonas aeruginosa*. Picosecond laser pulses of 780 nm were used to excite protein molecules. After a defined time-delay, the sample was probed by polychromatic x-ray pulses. Kinetic analysis of the experimental data revealed three structural intermediates, assigned as lumi-F, meta-F, and Pr, which are connected by time constants of 95 *μ*s and 21 ms. Molecular dynamics simulations show that the light activation of PaBphP promotes quaternary structural rearrangements from an “II”-framed close form of the Pfr state to an “O”-framed open form of the Pr state in terms of the helical backbones. The nearly parallel backbones in an II-framed close form become fully open with the O-framed geometry in the Pr state.

Another example of TRXSS studies conducted at BioCARS used T-jump approach to follow disordered stated in unfolding of calcium-bound bovine α-lactalbumin BLA.^124^ It is known that protein folding often executes through series of intermediate transient states which are partially folded. Understanding mechanism and process of partial folding for such states are very important but remains challenging because of disordered nature of such intermediates. In this experiment, 7 ns IR laser pulses were applied to BLA solution, and intermediate unfolded states were tracked by short polychromatic x-ray pulses. The experiments were conducted at 60 °C, 65 °C, and 70 °C. The unfolding process was tracked between 20 *μ*s and 70 ms and showed three distinct kinetic stated which can be attributed to partially disordered states of BLA protein. MD-simulations, constrained by TRXSS shown that one intermediate state is a molten globule state, while two lates states are terminal unfolded states. The kinetic model shows two parallel pathways: (1) folded state → I → U_1_ and (2) folded state- > U_2_. Calculated free energy surfaces (FES) of I, U_1_ and U_2_ identify I state as an intermediate, while U_1_ and U_2_ as an unfolded states with higher radius of gyration values and lower α-helix scores. The sampled structures of molten globular state I have similar overall topology, as folded BLA-protein but less condensed secondary structures, while U_1_ and U_2_ are much more conformally disordered and have expanded sizes. The results from this work were able to decipher protein folding process with a higher accuracy, than TRXSS or MD-simulations alone.

## APS UPGRADE IMPACT ON BioCARS

The APS is currently undergoing an extensive upgrade to replace its original electron storage ring with a new, state-of-the-art multi-bend achromat lattice. When the APS comes back online, many electron beam parameters will be different, and this will directly affect the time-resolved program at BioCARS.

The pulse duration of a single x-ray pulse in the current APS configuration is ∼100 ps (FWHM). After the APS upgrade, the pulse duration will extend to ∼250 ps. So, the best time resolution that BioCARS will be achievable after the upgrade is 250 ps. Finally, the APS storage ring current will increase from 100 to 200 mA. For all exposure times, except for single x-ray pulse exposures, x-ray flux will, therefore, be two times higher after the APS upgrade.

Another significant change for BioCARS is the change in the electron filling mode from the current standard 24-bunch mode of the storage ring to 48-bunch mode. This reduces the time between x-ray pulses in this mode from current ∼153 to ∼76 ns. As mentioned above, single pulse isolation is achieved by using BioCARS ultra-fast Jülich chopper designed specifically for the 24-bunch mode. Isolation of a single pulse in 48-bunch mode requires a change to the shape of the tunnel on the rotor of the chopper. A new upgraded chopper is expected to be available for resumed user operation in the summer of 2024.

The upgrade also eliminates the hybrid mode of the storage ring operation. This mode provided four times higher flux per single pulse than 24-bunch mode. In addition to the 48-bunch mode, the APS will provide a 324-bunch mode which has electron bunches that are 2.8 ns apart. The minimum opening time for the upgraded Jülich chopper will be ∼100 ns, so experiments that require time resolution of ∼100 ns or longer can utilize the 324-bunch mode.

A significant benefit of the multi-bend achromat lattice is a reduction in the horizontal beam size and divergence. This directly affects the final horizontal focusing at the sample position. The high divergence of the original lattice was the dominating factor in how small horizontal focus could be achieved at BioCARS. With upgrade, the slope errors on the horizontal mirrors become the limiting factor and the final horizontal focus is expected to be less than 10 *μ*m with the current focusing optics. The vertical beam size and divergence are not changing significantly so the final vertical focus should not change.

The changes in the overall operating parameters after the APS upgrade mean that our current undulators will have to be changed. The U23 and U27 undulators will be replaced with two U21 undulators. This mainly reduces the operating energy range from ∼6.5–19  to ∼8.5–15 keV but most of our experiments are done at 12 keV. Peak intensity and bandpass in a single x-ray pulse in 48 bunch mode at 12 keV are expected to be comparable to the current ones in 24-bunch mode but the total integrated flux will be slightly less. The main benefit of the upgrade is the ability to adjust the bandpass without reducing the peak intensity. Some experiments benefit from having a narrower bandpass. With the current setup, we can reduce it from 5% to 3% by reducing the white beam slits but this also reduces the peak intensity by ∼40%. With the combination of both the reduced horizontal divergence and new undulators, we expect that the bandpass can be reduced by the slits to 3% but without reducing the peak intensity.

In addition to the necessary upgrade of the ultra-fast Jülich chopper, BioCARS is also planning upgrade of the primary mirrors, adding a multilayer monochromator, and providing a vacuum chamber for the secondary KB mirrors.

Replacing the primary mirrors will allow for smaller focal spot sizes which is beneficial for some samples. This will also make it easier to isolate single pulses with the Jülich chopper and improve the overall beam stability. In addition, the new mirror systems will also have multi-point benders which allow for better control of the beam shape at the sample position. The current mirrors have a single point bender which produces a Gaussian peak only at the focus. A larger x-ray beam is sometimes desirable, but this typically results in a non-Gaussian peak shape at the sample position. The multi-point benders will fix this issue. After replacing the mirrors, the x-ray beam size is expected to be 10 × 5 *μ*m^2^ (V × H).

Laue experiments utilize the polychromatic beam at the 14-ID beamline. The current wide bandwidth (∼5%) results in higher polychromatic background and also results in elongated diffractions spots on samples with high mosaicity. Some experiments would, therefore, benefit from a narrower bandwidth (∼1%–2%). As mentioned above, after the APS upgrade 3% bandwidth will be achievable without a major reduction in peak intensity by reducing the white beam slit size. To achieve an even narrower bandwidth and still maintain a high peak intensity a multilayer monochromator is necessary. We, therefore, plan to replace the current Kohzu monochromator with a Double Crystal/Multilayer Monochromator (DCMM). With the new monochromator design, it will be easy to switch between standard monochromatic setup, a multilayer or to remove the optics from the beam entirely to still use the full polychromatic beam capabilities of the beamline. A cryo-cooled Si111 will provide tunability from 8.5–15 keV. The multilayer will be fixed at 12 keV which is where we are optimized to peak intensity.

The secondary KB mirror system provides a convenient way to adjust the final focus at the sample position. This works well for controlling the beam size, but the quality of the mirror surface can introduce additional background scatter. This is especially a concern with solution scattering experiments where a low background is required. The helium environment significantly reduces the contamination of the mirror surface, but small amounts of oxygen are still present and, in combination with the high intensity x-ray pulses, reduces the mirror quality. Placing the mirrors into a vacuum environment will fix the problem and increase the quality of the solution scattering data.

## CONCLUDING REMARKS

As envisioned by Keith Moffat decades ago, the central mission of BioCARS is to facilitate studies of dynamics in biological macromolecules. This objective is in line with broad needs of the structural biology community to move beyond structure determination to elucidating the internal mechanics that defines biochemical function in proteins and other complex macromolecules. The facility provides unique technical capabilities that open up new approaches for visualizing macromolecular dynamics at atomic resolutions and over biologically relevant timescales. We believe that the recent advances to streamline and simplify data collection and analysis will encourage the broad use of BioCARS by the structural biology community. Important scientific results of BioCARS user community over the years demonstrate the essential role that synchrotron facilities play in time-resolved structural studies, on time scales from 100 ps to milliseconds and longer. That role will continue well into the future with recent or ongoing major upgrades of many synchrotrons, including the Advanced Photon Source, that are significantly improving the brilliance of these x-ray source.

## Data Availability

Data sharing is not applicable to this article as no new data were created or analyzed in this study.

## References

[1] B. Alberts , “ The cell as a collection of protein machines: Preparing the next generation of molecular biologists,” Cell 92, 291–294 (1998). 10.1016/S0092-8674(00)80922-8 9476889

[2] J. Méndez and B. Stillman , “ Perpetuating the double helix: Molecular machines at eukaryotic DNA replication origins,” BioEssays 25, 1158–1167 (2003). 10.1002/bies.10370 14635251

[3] D. D. Boehr , D. McElheny , H. J. Dyson , and P. E. Wright , “ The dynamic energy landscape of dihydrofolate reductase catalysis,” Science 313, 1638–1642 (2006). 10.1126/science.1130258 16973882

[4] H. Noji , R. Yasuda , M. Yoshida , and K. Kinosita , “ Direct observation of the rotation of F1-ATPase,” Nature 386, 299–302 (1997). 10.1038/386299a0 9069291

[5] H. Krishnamurthy and E. Gouaux , “ X-ray structures of LeuT in substrate-free outward-open and apo inward-open states,” Nature 481, 469–474 (2012). 10.1038/nature10737 22230955 PMC3306218

[6] R. D. Vale and R. A. Milligan , “ The way things move: Looking under the hood of molecular motor proteins,” Science 288, 88–95 (2000). 10.1126/science.288.5463.88 10753125

[7] S. R. Sprang , “ G PROTEIN MECHANISMS: Insights from structural analysis,” Annu. Rev. Biochem. 66, 639–678 (1997). 10.1146/annurev.biochem.66.1.639 9242920

[8] M. Karplus and J. A. McCammon , “ Molecular dynamics simulations of biomolecules,” Nat. Struct. Biol. 9, 646–652 (2002). 10.1038/nsb0902-646 12198485

[9] W. J. Albery and J. R. Knowles , “ Efficiency and evolution of enzyme catalysis,” Angew. Chem. Int. Ed. Engl. 16, 285–293 (1977). 10.1002/anie.197702851 406815

[10] J. L. Knies , F. Cai , and D. M. Weinreich , “ Enzyme efficiency but not thermostability drives cefotaxime resistance evolution in TEM-1 β-lactamase,” Mol. Biol. Evol. 34, 1040–1054 (2017). 28087769 10.1093/molbev/msx053 PMC5400381

[11] B. K. Shoichet , W. A. Baase , R. Kuroki , and B. W. Matthews , “ A relationship between protein stability and protein function,” Proc. Natl. Acad. Sci. U. S. A. 92, 452–456 (1995). 10.1073/pnas.92.2.452 7831309 PMC42758

[12] N. Tokuriki and D. S. Tawfik , “ Protein dynamism and evolvability,” Science 324, 203–207 (2009). 10.1126/science.1169375 19359577

[13] N. Halabi , O. Rivoire , S. Leibler , and R. Ranganathan , “ Protein sectors: Evolutionary units of three-dimensional structure,” Cell 138, 774–786 (2009). 10.1016/j.cell.2009.07.038 19703402 PMC3210731

[14] R. K. Jain and R. Ranganathan , “ Local complexity of amino acid interactions in a protein core,” Proc. Natl. Acad. Sci. U. S. A. 101, 111–116 (2004). 10.1073/pnas.2534352100 14684834 PMC314147

[15] L. Hedstrom , “ Trypsin: A case study in the structural determinants of enzyme specificity,” Biol. Chem. 377, 465–470 (1996). 8922280

[16] U. Gether , “ Uncovering molecular mechanisms involved in activation of G protein-coupled receptors,” Endocr. Rev. 21, 90–113 (2000). 10.1210/edrv.21.1.0390 10696571

[17] M. F. Perutz , A. J. Wilkinson , M. Paoli , and G. G. Dodson , “ The stereochemical mechanism of the cooperative effects in hemoglobin revisited,” Annu. Rev. Biophys. Biomol. Struct. 27, 1–34 (1998). 10.1146/annurev.biophys.27.1.1 9646860

[18] S. J. Benkovic and S. Hammes-Schiffer , “ A perspective on enzyme catalysis,” Science 301, 1196–1202 (2003). 10.1126/science.1085515 12947189

[19] K. S. Midelfort and K. D. Wittrup , “ Context-dependent mutations predominate in an engineered high-affinity single chain antibody fragment,” Protein Sci. 15, 324–334 (2006). 10.1110/ps.051842406 16434745 PMC2242459

[20] P. A. Patten *et al.*, “ The immunological evolution of catalysis,” Science 271, 1086–1091 (1996). 10.1126/science.271.5252.1086 8599084

[21] T. Clackson and J. A. Wells , “ A hot spot of binding energy in a hormone-receptor interface,” Science 267, 383–386 (1995). 10.1126/science.7529940 7529940

[22] R. N. McLaughlin, Jr. , F. J. Poelwijk , A. Raman , W. S. Gosal , and R. Ranganathan , “ The spatial architecture of protein function and adaptation,” Nature 491, 138–142 (2012). 10.1038/nature11500 23041932 PMC3991786

[23] A. S. Raman , K. I. White , and R. Ranganathan , “ Origins of allostery and evolvability in proteins: A case study,” Cell 166, 468–480 (2016). 10.1016/j.cell.2016.05.047 27321669

[24] E. Z. Eisenmesser *et al.*, “ Intrinsic dynamics of an enzyme underlies catalysis,” Nature 438, 117–121 (2005). 10.1038/nature04105 16267559

[25] K. Henzler-Wildman and D. Kern , “ Dynamic personalities of proteins,” Nature 450, 964–972 (2007). 10.1038/nature06522 18075575

[26] K. A. Henzler-Wildman *et al.*, “ A hierarchy of timescales in protein dynamics is linked to enzyme catalysis,” Nature 450, 913–916 (2007). 10.1038/nature06407 18026087

[27] L. E. Kay , “ Protein dynamics from NMR,” Biochem. Cell Biol. 76, 145–152 (1998). 10.1139/o98-024 9923683

[28] A. Sekhar and L. E. Kay , “ NMR paves the way for atomic level descriptions of sparsely populated, transiently formed biomolecular conformers,” Proc. Natl. Acad. Sci. U. S. A. 110, 12867–12874 (2013). 10.1073/pnas.1305688110 23868852 PMC3740838

[29] K. C. Neuman and A. Nagy , “ Single-molecule force spectroscopy: Optical tweezers, magnetic tweezers and atomic force microscopy,” Nat. Methods 5, 491–505 (2008). 10.1038/nmeth.1218 18511917 PMC3397402

[30] K. Moffat , “ Time-resolved biochemical crystallography: A mechanistic perspective,” Chem. Rev. 101, 1569–1582 (2001). 10.1021/cr990039q 11709992

[31] A. L. Fink , D. Kar , and R. Kotin , “ Ribonuclease structure and catalysis: Effects of crystalline enzyme, alcohol cryosolvents, low temperatures, and product inhibition,” Biochemistry 26, 8571–8579 (1987). 10.1021/bi00400a012 3442677

[32] P. J. Kasvinsky and N. B. Madsen , “ Activity of glycogen phosphorylase in the crystalline state,” J. Biol. Chem. 251, 6852–6859 (1976). 10.1016/S0021-9258(17)33022-3 824288

[33] J. R. Kiefer , C. Mao , J. C. Braman , and L. S. Beese , “ Visualizing DNA replication in a catalytically active Bacillus DNA polymerase crystal,” Nature 391, 304–307 (1998). 10.1038/34693 9440698

[34] F. A. Quiocho , C. H. Mcmurray , and W. N. Lipscomb , “ Similarities between the conformation of arsanilazotyrosine 248 of carboxypeptidase Aα in the crystalline state and in solution,” Proc. Natl. Acad. Sci. U. S. A. 69, 2850–2854 (1972). 10.1073/pnas.69.10.2850 4507609 PMC389660

[35] I. Schlichting *et al.*, “ Time-resolved x-ray crystallographic study of the conformational change in Ha-Ras p21 protein on GTP hydrolysis,” Nature 345, 309–315 (1990). 10.1038/345309a0 2111463

[36] J. B. Sumner , “ The isolation and crystallization of the enzyme urease. Preliminary paper,” in *The Isolation and Crystallization of the Enzyme Urease. Preliminary Paper* ( Harvard University Press, 2013), pp. 322–327.

[37] C. Kupitz *et al.*, “ Structural enzymology using x-ray free electron lasers,” Struct. Dyn. 4, 044003 (2017). 10.1063/1.4972069 28083542 PMC5178802

[38] P. Mehrabi *et al.*, “ Time-resolved crystallography reveals allosteric communication aligned with molecular breathing,” Science 365, 1167–1170 (2019). 10.1126/science.aaw9904 31515393

[39] S. Mous *et al.*, “ Dynamics and mechanism of a light-driven chloride pump,” Science 375, 845–851 (2022). 10.1126/science.abj6663 35113649

[40] J. L. Olmos *et al.*, “ Enzyme intermediates captured “on the fly” by mix-and-inject serial crystallography,” BMC Biol. 16, 59 (2018). 10.1186/s12915-018-0524-5 29848358 PMC5977757

[41] D. Sorigué *et al.*, “ Mechanism and dynamics of fatty acid photodecarboxylase,” Science 372, eabd5687 (2021). 10.1126/science.abd5687 33833098

[42] M. Wilamowski *et al.*, “ Time-resolved β-lactam cleavage by L1 metallo-β-lactamase,” Nat. Commun. 13, 7379 (2022). 10.1038/s41467-022-35029-3 36450742 PMC9712583

[43] G. Brändén and R. Neutze , “ Advances and challenges in time-resolved macromolecular crystallography,” Science 373, eaba0954 (2021). 10.1126/science.aba0954 34446579

[44] D. R. Hekstra , “ Emerging time-resolved x-ray diffraction approaches for protein dynamics,” Annu. Rev. Biophys. 52, 255–274 (2023). 10.1146/annurev-biophys-111622-091155 37159292 PMC10687665

[45] T. N. Malla and M. Schmidt , “ Transient state measurements on proteins by time-resolved crystallography,” Curr. Opin. Struct. Biol. 74, 102376 (2022). 10.1016/j.sbi.2022.102376 35468535

[46] M. A. Wilson , “ Mapping enzyme landscapes by time-resolved crystallography with synchrotron and x-ray free electron laser light,” Annu. Rev. Biophys. 51, 79–98 (2022). 10.1146/annurev-biophys-100421-110959 34932909 PMC9132212

[47] C. A. Brosey and J. A. Tainer , “ Evolving SAXS versatility: Solution x-ray scattering for macromolecular architecture, functional landscapes, and integrative structural biology,” Curr. Opin. Struct. Biol. 58, 197–213 (2019). 10.1016/j.sbi.2019.04.004 31204190 PMC6778498

[48] N. M. Kirby and N. P. Cowieson , “ Time-resolved studies of dynamic biomolecules using small angle x-ray scattering,” Curr. Opin. Struct. Biol. 28, 41–46 (2014). 10.1016/j.sbi.2014.07.007 25108308

[49] K. Moffat , “ Time-resolved macromolecular crystallography,” Annu. Rev. Biophys. Biophys. Chem. 18, 309–332 (1989). 10.1146/annurev.bb.18.060189.001521 2660828

[50] K. Moffat , D. Szebenyi , and D. Bilderback , “ X-ray Laue diffraction from protein crystals,” Science 223, 1423–1425 (1984). 10.1126/science.223.4643.1423 17746054

[51] K. Moffat and J. R. Helliwell , “ The Laue method and its use in time-resolved crystallography,” in *Synchrotron Radiation in Chemistry and Biology III*, edited by E. Mandelkow ( Springer, 1989), pp. 61–74.

[52] K. Moffat , D. Bilderback , W. Schildkamp , and K. Volz , “ Laue diffraction from biological samples,” Nucl. Instrum. Methods Phys. Res., Sect. A 246, 627–635 (1986). 10.1016/0168-9002(86)90164-6

[53] D. Bourgeois *et al.*, “ Feasibility and realization of single-pulse Laue diffraction on macromolecular crystals at ESRF,” J. Synchrotron Radiat. 3, 65–74 (1996). 10.1107/S090904959501661X 16702661

[54] V. Šrajer *et al.*, “ Photolysis of the carbon monoxide complex of myoglobin: Nanosecond time-resolved crystallography,” Science 274, 1726–1729 (1996). 10.1126/science.274.5293.1726 8939867

[55] U. K. Genick *et al.*, “ Structure of a protein photocycle intermediate by millisecond time-resolved crystallography,” Science 275, 1471–1475 (1997). 10.1126/science.275.5305.1471 9045611

[56] B. Perman *et al.*, “ Energy transduction on the nanosecond time scale: Early structural events in a xanthopsin photocycle,” Science 279, 1946–1950 (1998). 10.1126/science.279.5358.1946 9506946

[57] Z. Ren *et al.*, “ A molecular movie at 1.8 Å resolution displays the photocycle of photoactive yellow protein, a Eubacterial blue-light receptor, from nanoseconds to seconds,” Biochemistry 40, 13788–13801 (2001). 10.1021/bi0107142 11705368

[58] V. Šrajer *et al.*, “ Protein conformational relaxation and ligand migration in myoglobin: A nanosecond to millisecond molecular movie from time-resolved laue x-ray diffraction,” Biochemistry 40, 13802–13815 (2001). 10.1021/bi010715u 11705369

[59] M. Schmidt *et al.*, “ Protein kinetics: Structures of intermediates and reaction mechanism from time-resolved x-ray data,” Proc. Natl. Acad. Sci. U. S. A. 101, 4799–4804 (2004). 10.1073/pnas.0305983101 15041745 PMC387328

[60] S. Anderson *et al.*, “ Chromophore conformation and the evolution of tertiary structural changes in photoactive yellow protein,” Structure 12, 1039–1045 (2004). 10.1016/j.str.2004.04.008 15274923

[61] H. Ihee *et al.*, “ Visualizing reaction pathways in photoactive yellow protein from nanoseconds to seconds,” Proc. Natl. Acad. Sci. U. S. A. 102, 7145–7150 (2005). 10.1073/pnas.0409035102 15870207 PMC1088170

[62] M. Schmidt *et al.*, “ Ligand migration pathway and protein dynamics in myoglobin: A time-resolved crystallographic study on L29W MbCO,” Proc. Natl. Acad. Sci. U. S. A. 102, 11704–11709 (2005). 10.1073/pnas.0504932102 16085709 PMC1187994

[63] J. E. Knapp , R. Pahl , V. Šrajer , and W. E. Royer , “ Allosteric action in real time: Time-resolved crystallographic studies of a cooperative dimeric hemoglobin,” Proc. Natl. Acad. Sci. U. S. A. 103, 7649–7654 (2006). 10.1073/pnas.0509411103 16684887 PMC1472499

[64] J. Key , V. Šrajer , R. Pahl , and K. Moffat , “ Time-resolved crystallographic studies of the heme domain of the oxygen sensor FixL: structural dynamics of ligand rebinding and their relation to signal transduction,” Biochemistry 46, 4706–4715 (2007). 10.1021/bi700043c 17385895

[65] J. E. Knapp *et al.*, “ Ligand migration and cavities within *Scapharca* dimeric HbI: Studies by time-resolved crystallography, Xe binding and computational analysis,” Structure 17, 1494–1504 (2009). 10.1016/j.str.2009.09.004 19913484 PMC2785043

[66] M. Schmidt , T. Graber , R. Henning , and V. Srajer , “ Five-dimensional crystallography,” Acta Crystallogr., Sect. A: Found. Crystallogr. 66, 198–206 (2010). 10.1107/S0108767309054166 PMC2824529 20164643

[67] T. Graber *et al.*, “ BioCARS: A synchrotron resource for time-resolved x-ray science,” J. Synchrotron Radiat. 18, 658–670 (2011). 10.1107/S0909049511009423 21685684 PMC3121234

[68] H. S. Cho *et al.*, “ Protein structural dynamics in solution unveiled via 100-ps time-resolved x-ray scattering,” Proc. Natl. Acad. Sci. U. S. A. 107, 7281–7286 (2010). 10.1073/pnas.1002951107 20406909 PMC2867760

[69] K. M. Dalton , J. B. Greisman , and D. R. Hekstra , “ A unifying Bayesian framework for merging x-ray diffraction data,” Nat. Commun. 13, 7764 (2022). 10.1038/s41467-022-35280-8 36522310 PMC9755530

[70] Y. Gevorkov *et al.*, “ pinkIndexer—A universal indexer for pink-beam x-ray and electron diffraction snapshots,” Acta Crystallogr., Sect. A: Found. Adv. 76, 121–131 (2020). 10.1107/S2053273319015559 32124850 PMC7053222

[71] J. B. Greisman , K. M. Dalton , and D. R. Hekstra , “ reciprocalspaceship: A Python library for crystallographic data analysis,” J. Appl. Crystallogr. 54, 1521–1529 (2021). 10.1107/S160057672100755X 34671231 PMC8493618

[72] J. R. Stagno , Y. R. Bhandari , C. E. Conrad , Y. Liu , and Y.-X. Wang , “ Real-time crystallographic studies of the adenine riboswitch using an x-ray free-electron laser,” FEBS J. 284, 3374–3380 (2017). 10.1111/febs.14110 28504865 PMC6309305

[73] S. Pandey *et al.*, “ Observation of substrate diffusion and ligand binding in enzyme crystals using high-repetition-rate mix-and-inject serial crystallography,” IUCrJ 8, 878–895 (2021). 10.1107/S2052252521008125 PMC8562667 34804542

[74] T. N. Malla *et al.*, “ Heterogeneity in *M. tuberculosis* β-lactamase inhibition by Sulbactam,” Nat. Commun. 14, 5507 (2023). 10.1038/s41467-023-41246-1 37679343 PMC10485065

[75] T. A. White *et al.*, “ Crystallographic data processing for free-electron laser sources,” Acta Crystallogr., Sect. D: Biol. Crystallogr. 69, 1231–1240 (2013). 10.1107/S0907444913013620 23793149 PMC3689526

[76] I. Schlichting , “ Serial femtosecond crystallography: The first five years,” IUCrJ 2, 246–255 (2015). 10.1107/S205225251402702X PMC4392417 25866661

[77] S. Boutet *et al.*, “ High-resolution protein structure determination by serial femtosecond crystallography,” Science 337, 362–364 (2012). 10.1126/science.1217737 22653729 PMC3788707

[78] F. Stellato *et al.*, “ Room-temperature macromolecular serial crystallography using synchrotron radiation,” IUCrJ 1, 204–212 (2014). 10.1107/S2052252514010070 PMC4107920 25075341

[79] J. Tenboer *et al.*, “ Time-resolved serial crystallography captures high-resolution intermediates of photoactive yellow protein,” Science 346, 1242–1246 (2014). 10.1126/science.1259357 25477465 PMC4361027

[80] U. Weierstall *et al.*, “ Lipidic cubic phase injector facilitates membrane protein serial femtosecond crystallography,” Nat. Commun. 5, 3309 (2014). 10.1038/ncomms4309 24525480 PMC4061911

[81] P. Nogly *et al.*, “ Lipidic cubic phase serial millisecond crystallography using synchrotron radiation,” IUCrJ 2, 168–176 (2015). 10.1107/S2052252514026487 PMC4392771 25866654

[82] K. Pande *et al.*, “ Femtosecond structural dynamics drives the trans/cis isomerization in photoactive yellow protein,” Science 352, 725–729 (2016). 10.1126/science.aad5081 27151871 PMC5291079

[83] K. Diederichs and M. Wang , “ Serial synchrotron x-ray crystallography (SSX),” in *Protein Crystallography: Methods and Protocols*, edited by A. Wlodawer , Z. Dauter , and M. Jaskolski ( Springer, 2017), pp. 239–272. 10.1007/978-1-4939-7000-1_10 28573576

[84] J. M. Martin-Garcia *et al.*, “ Serial millisecond crystallography of membrane and soluble protein microcrystals using synchrotron radiation,” IUCrJ 4, 439–454 (2017). 10.1107/S205225251700570X PMC5571807 28875031

[85] Z. Ren *et al.*, “ Laue crystallography: Coming of age,” J. Synchrotron Radiat. 6, 891–917 (1999). 10.1107/S0909049599006366

[86] A. Meents *et al.*, “ Pink-beam serial crystallography,” Nat. Commun. 8, 1281 (2017). 10.1038/s41467-017-01417-3 29097720 PMC5668288

[87] J. M. Martin-Garcia *et al.*, “ High-viscosity injector-based pink-beam serial crystallography of microcrystals at a synchrotron radiation source,” IUCrJ 6, 412–425 (2019). 10.1107/S205225251900263X PMC6503920 31098022

[88] J. Lieske *et al.*, “ On-chip crystallization for serial crystallography experiments and on-chip ligand-binding studies,” IUCrJ 6, 714–728 (2019). 10.1107/S2052252519007395 PMC6608620 31316815

[89] D. A. Sherrell *et al.*, “ Fixed-target serial crystallography at the Structural Biology Center,” J. Synchrotron Radiat. 29, 1141–1151 (2022). 10.1107/S1600577522007895 36073872 PMC9455217

[90] M. Carrillo *et al.*, “ High-resolution crystal structures of transient intermediates in the phytochrome photocycle,” Structure 29, 743–754 (2021). 10.1016/j.str.2021.03.004 33756101 PMC8405169

[91] M. Schmidt , “ Mix and inject: Reaction initiation by diffusion for time-resolved macromolecular crystallography,” Adv. Condens. Matter Phys. 2013, e167276.

[92] G. D. Calvey , A. M. Katz , and L. Pollack , “ Microfluidic mixing injector holder enables routine structural enzymology measurements with mix-and-inject serial crystallography using x-ray free electron lasers,” Anal. Chem. 91, 7139–7144 (2019). 10.1021/acs.analchem.9b00311 31060352

[93] G. D. Calvey , A. M. Katz , C. B. Schaffer , and L. Pollack , “ Mixing injector enables time-resolved crystallography with high hit rate at x-ray free electron lasers,” Struct. Dyn. 3, 054301 (2016). 10.1063/1.4961971 27679802 PMC5010557

[94] D. R. Hekstra *et al.*, “ Electric-field-stimulated protein mechanics,” Nature 540, 400–405 (2016). 10.1038/nature20571 27926732 PMC5730412

[95] A. M. Wolff *et al.*, “ Mapping protein dynamics at high spatial resolution with temperature-jump x-ray crystallography,” Nat. Chem. 15, 1549–1558 (2023). 10.1038/s41557-023-01329-4 37723259 PMC10624634

[96] Z. Ren *et al.*, “ An automated platform for *in situ* serial crystallography at room temperature,” IUCrJ 7, 1009–1018 (2020). 10.1107/S2052252520011288 PMC7642789 33209315

[97] S. Bandara *et al.*, “ Crystal structure of a far-red–sensing cyanobacteriochrome reveals an atypical bilin conformation and spectral tuning mechanism,” Proc. Natl. Acad. Sci. U. S. A. 118, e2025094118 (2021). 10.1073/pnas.2025094118 33727422 PMC8000052

[98] L. M. Biju *et al.*, “ On-chip crystallization and large-scale serial diffraction at room temperature,” J. Vis. Exp. 2022, e63022. 10.3791/63022 35343951

[99] J. B. Greisman *et al.*, “ Resolving conformational changes that mediate a two-step catalytic mechanism in a model enzyme,” bioRxiv:2023.06.02.543507 (2023).

[100] A. S. Pawate *et al.*, “ Towards time-resolved serial crystallography in a microfluidic device,” Acta Crystallogr., Sect. F: Struct. Biol. Commun. 71, 823–830 (2015). 10.1107/S2053230X15009061 26144226 PMC4498702

[101] G. Winter *et al.*, “ DIALS as a toolkit,” Protein Sci. 31, 232–250 (2022). 10.1002/pro.4224 34747533 PMC8740827

[102] T. A. White *et al.*, “ CrystFEL: A software suite for snapshot serial crystallography,” J. Appl. Crystallogr. 45, 335–341 (2012). 10.1107/S0021889812002312

[103] A. Tolstikova *et al.*, “ 1 kHz fixed-target serial crystallography using a multilayer monochromator and an integrating pixel detector,” IUCrJ 6, 927 (2019). 10.1107/S205225251900914X PMC6760437 31576225

[104] K. Nass *et al.*, “ Pink-beam serial femtosecond crystallography for accurate structure-factor determination at an X-ray free-electron laser,” IUCrJ 8, 905–920 (2021). 10.1107/S2052252521008046 PMC8562661 34804544

[105] A. Tolstikova , *Development of Diffraction Analysis Methods for Serial Crystallography* ( University of Hamburg, 2020).

[106] C. D. Putnam , M. Hammel , G. L. Hura , and J. A. Tainer , “ X-ray solution scattering (SAXS) combined with crystallography and computation: Defining accurate macromolecular structures, conformations and assemblies in solution,” Q. Rev. Biophys. 40, 191–285 (2007). 10.1017/S0033583507004635 18078545

[107] E. E. Lattman , T. D. Grant , and E. H. Snell , *Biological Small Angle Scattering: Theory and Practice* ( Oxford University Press, 2018).

[108] K. H. Kim *et al.*, “ Direct observation of myoglobin structural dynamics from 100 picoseconds to 1 microsecond with picosecond X-ray solution scattering,” Chem. Commun. 47, 289–291 (2011). 10.1039/C0CC01817A PMC2999690 20733999

[109] K. H. Kim *et al.*, “ Direct observation of cooperative protein structural dynamics of homodimeric hemoglobin from 100 ps to 10 ms with pump–probe x-ray solution scattering,” J. Am. Chem. Soc. 134, 7001–7008 (2012). 10.1021/ja210856v 22494177 PMC3337689

[110] H. S. Cho *et al.*, “ Picosecond photobiology: Watching a signaling protein function in real time via time-resolved small- and wide-angle x-ray scattering,” J. Am. Chem. Soc. 138, 8815–8823 (2016). 10.1021/jacs.6b03565 27305463 PMC5336379

[111] D. J. Hsu *et al.*, “ X-ray snapshots reveal conformational influence on active site ligation during metalloprotein folding,” Chem. Sci. 10, 9788–9800 (2019). 10.1039/C9SC02630D 32055348 PMC6993610

[112] A. M. Chan *et al.*, “ The role of transient intermediate structures in the unfolding of the Trp-cage fast-folding protein: Generating ensembles from time-resolved x-ray solution scattering with genetic algorithms,” J. Phys. Chem. Lett. 14, 1133–1139 (2023). 10.1021/acs.jpclett.2c03680 36705525 PMC10167713

[113] M. C. Thompson *et al.*, “ Temperature-jump solution x-ray scattering reveals distinct motions in a dynamic enzyme,” Nat. Chem. 11, 1058–1066 (2019). 10.1038/s41557-019-0329-3 31527847 PMC6815256

[114] A. L. Bennett *et al.*, “ Microsecond dynamics control the HIV-1 envelope conformation,” bioRxiv 2023.05.17.541130 (2023). 10.1126/sciadv.adj0396 PMC10836732 38306419

[115] D. Rimmerman *et al.*, “ Probing cytochrome c folding transitions upon phototriggered environmental perturbations using time-resolved x-ray scattering,” J. Phys. Chem. B 122, 5218–5224 (2018). 10.1021/acs.jpcb.8b03354 29709179 PMC6503524

[116] H. S. Cho *et al.*, “ Dynamics of quaternary structure transitions in r-state carbonmonoxyhemoglobin unveiled in time-resolved x-ray scattering patterns following a temperature jump,” J. Phys. Chem. B 122, 11488–11496 (2018). 10.1021/acs.jpcb.8b07414 30285440 PMC6580858

[117] T. W. Kim *et al.*, “ Protein folding from heterogeneous unfolded state revealed by time-resolved X-ray solution scattering,” Proc. Natl. Acad. Sci. U. S. A. 117, 14996–15005 (2020). 10.1073/pnas.1913442117 32541047 PMC7334511

[118] S. J. Lee *et al.*, “ Light-induced protein structural dynamics in bacteriophytochrome revealed by time-resolved x-ray solution scattering,” Sci. Adv. 8, eabm6278 (2022). 10.1126/sciadv.abm6278 35622911 PMC9140987

[119] D. Rimmerman *et al.*, “ Direct observation of insulin association dynamics with time-resolved x-ray scattering,” J. Phys. Chem. Lett. 8, 4413–4418 (2017). 10.1021/acs.jpclett.7b01720 28853898 PMC5804350

[120] I. Kosheleva *et al.*, “ Sample-minimizing co-flow cell for time-resolved pump–probe x-ray solution scattering,” J. Synchrotron Radiat. 30, 490–499 (2023). 10.1107/S1600577522012127 36891863 PMC10000795

[121] E. B. Knudsen , H. O. Sørensen , J. P. Wright , G. Goret , and J. Kieffer , “ FabIO: Easy access to two-dimensional x-ray detector images in Python,” J. Appl. Crystallogr. 46, 537–539 (2013). 10.1107/S0021889813000150

[122] G. Ashiotis *et al.*, “ The fast azimuthal integration Python library: PyFAI,” J. Appl. Crystallogr. 48, 510–519 (2015). 10.1107/S1600576715004306 25844080 PMC4379438

[123] P. V. Konarev , M. V. Petoukhov , V. V. Volkov , and D. I. Svergun , “ ATSAS 2.1, a program package for small-angle scattering data analysis,” J. Appl. Crystallogr. 39, 277–286 (2006). 10.1107/S0021889806004699 PMC4233345 25484842

[124] D. J. Hsu , D. Leshchev , I. Kosheleva , K. L. Kohlstedt , and L. X. Chen , “ Unfolding bovine α-lactalbumin with T-jump: Characterizing disordered intermediates via time-resolved x-ray solution scattering and molecular dynamics simulations,” J. Chem. Phys. 154, 105101 (2021). 33722011 10.1063/5.0039194 PMC7943248

